# When describing harms and benefits to potential trial participants, participant information leaflets are inadequate

**DOI:** 10.1186/s13063-024-08087-9

**Published:** 2024-05-01

**Authors:** Laura Cuddihy, Jeremy Howick, Ellen Murphy, Frances Shiely

**Affiliations:** 1 Oncology Day Ward, St. James’s Hospital, James Street, Dublin 8, Ireland; 2 Trials Research and Methodologies Unit (TRAMS), HRB Clinical Research Facility and School of Public Health, 4th Floor Western Gateway Building, University College Cork, Cork, Ireland; 3 Medical School, Stoneygate Centre for Excellence in Empathic Healthcare, University of Leicester, George Davies Centre, Lancaster Rd., Leicester, LE1 7HA UK; 4 School of Public Health, University College Cork, Cork, Ireland

**Keywords:** Trial methodology, Risks, Benefits and harms, Patient information leaflets, Participant information leaflets, Informed consent

## Abstract

**Background:**

Providing informed consent for trials requires providing trial participants with comprehensive information about the trial, including information about potential risks and benefits. It is required by the ethical principle of respecting patient autonomy. Our study examines the variation in the way information about potential trial benefits and harms is shared in participant information leaflets (PILs).

**Methods:**

A total of 214 PILs and informed consent forms from clinical trials units (CTUs) and Clinical Research Facilities (CRFs) in Ireland and the UK were assessed by two authors independently, to check the extent to which they adhered to seven recently developed principles. Discrepancies were resolved by a third.

**Results:**

Usage of the seven principles varied widely between PILs regardless of the intended recipient or trial type. None of the PILs used more than four principles, and some (4%) used none. Twenty-seven per cent of PILs presented information about all known potential harms, whereas 45% presented information on all known potential benefits. Some PILs did not provide any potential harms or potential benefits (8%). There was variation in the information contained in adult and children PILs and across disease areas.

**Conclusion:**

Significant variation exists in how potential trial benefits and harms are described to potential trial participants in PILs in our sample. Usage of the seven principles of good practice will promote consistency, ensure informed ethical decision-making and invoke trust and transparency. In the long term, a standardised PIL template is needed.

**Supplementary Information:**

The online version contains supplementary material available at 10.1186/s13063-024-08087-9.

## Background

Randomised trials are conducted to provide evidence to support better and more informed decisions to improve patient choice and care [1]. The informed consent process for trials aims to provide patients with comprehensive information about the trial, including potential risks and benefits [2] and is required by the ethical principle of respecting patient autonomy stated in the Nuremberg Code [3, 4] and the Declaration of Helsinki [5, 6]. To assist in making an informed decision about participating in a trial, participants are provided with a participant information leaflet (PIL) which is a requirement of the International Conference on Harmonisation Good Clinical Practice (ICH GCP) [7, 8].

The UK Health Research Authority (HRA) provide guidance on the process of developing PILs and including informed consent forms [9]. Their website provides a template with headings with general recommendations. For example, in the section, “what are the disadvantages or risks of taking part”, the template states, “You should include details of all significant risks of harm, risks to confidentiality and psychological risk. Some specific issues you should consider include: Impact on possible pregnancy and breast feeding, including young people and pregnancy; Side effects of treatments/therapies in trials; Discovering health related findings; Impact on insurance; Ionising radiation etc. Try to describe the likelihood of adverse things happening, as well as severity in language all potential participants are likely to understand”. However, this advice appears to be ambiguous to researchers, as a previous study has shown that the current HRA guidance is not applied consistently and is sometimes ignored [10].

The way the information within the PIL is presented to patients has consequences. For example, it can influence participants’ expectations and subsequently impact their experiences in the trial [11, 12]. Over-emphasising potential harms can heighten participants’ concerns and increase the likelihood of experiencing nocebo effects, a phenomenon where participants experience adverse effects or worsening of symptoms due to negative expectations or beliefs about a treatment [11, 13]. Several studies report the nocebo effect, where participants allocated to the placebo group experienced adverse effects, sometimes in excess of 50% of trial participants [14–19]. Paradoxically, the opposite can also be true: without being provided with sound evidence about potential benefits, participants can overestimate potential benefits, a phenomenon known as therapeutic optimism [20, 21].

Researchers and healthcare professionals have an ethical responsibility to provide accurate and balanced information to trial participants [16]. It is important to disclose potential benefits and harms in a transparent manner while avoiding unnecessary fear-inducing language or exaggerating descriptions of side effects. Striking the right balance is crucial to ensure that participants are fully informed without inducing undue anxiety or nocebo effects [22]. Despite the ethical requirement to provide accurate information about potential harms and potential benefits, a recent study of 33 PILs found that while most contain complete information about potential harms, many do not mention potential benefits at all [10]. This might not be surprising since guidance on how to effectively design a PIL is lacking [23] with much variation evident between PILs of different trial types, disease areas etc. [12].

However, the extent of the inconsistency in the way information about potential benefits and harms is shared within PILs is not known; examining a larger sample of PILs is required to establish this. A method for reducing the variation in PILs was recently developed in the context of the Medical Research Council (MRC)-funded ‘PrinciPILs’ project [24]. The project produced 7 principles to guide how information about potential benefits and harms should be conveyed within PILs. The principles were developed with input from over 200 stakeholders from the pharmaceutical industry, trial managers, research ethics committee members and patient representatives who participated in a modified Delphi process and subsequent consensus meeting [25]. The principles are as follows: (1) all potential harms of an intervention should be listed, (2) all potential harms should be divided into serious (life-threatening, causing permanent damage) and less serious (like a mild headache that goes away quickly), (3) it must be made explicit that not all potential harms are known, (4) all potential benefits of the intervention should be listed, (5) all potential benefits and harms need to be compared with what would happen if the participant did not take part in the trial, (6) suitable visual representations are recommended where appropriate to describe potential intervention benefits and harms, and (7) information regarding potential benefits and harms should not be presented apart by one or more pages [25].

The purpose of our study was to examine 214 PILs to examine the variations in the way information about potential benefits and harms is conveyed by using the 7 recently developed principles as a frame of reference.

## Methods

### Data collection

We previously collected PILs and ICFs for another study [26]. To obtain these, clinical trial units (CTUs)/Clinical Research Facilities (CRFs) in the UK and Ireland were contacted to provide us with PILs/ICFs and any other materials used when recruiting participants to randomised controlled trials (RCTs). We requested all available PILs from their respective repositories. The inclusion criteria were RCTs at any phase, cohort studies with an embedded RCT, feasibility studies, pilot studies and studies conducted in any language and from any year. Adult PILs, PILs provided to parents whose children were being recruited, PILs for family members or legal representatives of those who did not have the capacity to consent and PILs for those who regained the capacity to consent were included. Some studies had both a PIL and an ICF. If the information contained in both the ICF and the PIL was duplicated, only the PIL was included. Combined PIL/ICFs were included, but only information from the PIL was analysed.

Data for this study was independently extracted by two authors. Variables extracted were as follows: study name, year the PIL was created, study design, phase of the study, funding sources, clinical speciality, planned sample size, study population, intervention and comparator, follow-up information, primary outcome and usage of the seven principles. An initial training meeting was held between all authors to discuss the data extraction process. JH, the principal investigator of the PrinciPILs project, provided the training using examples from PILs not included in this project. Based on this, LC piloted the data extraction on five PILs, and this was double extracted by EM. There were some small discrepancies which were resolved by discussion with a third reviewer, FS. JH reviewed these five extractions and agreed with all the extractions. We piloted another five using the same methodology and agreement was reached on all five. The remaining extractions were conducted by LC (*n* = 204), and EM completed a double extraction on a random 10% sample (*n* = 21). Discrepancies (< 1%) were discussed between LC and EM. When assessing if the PIL met principle 1, present all harms, and principle 4, present all benefits, additional information on the study intervention was necessary to make this judgement. This was obtained from the Summary of Product Characteristics (SpC) for clinical trials of an investigational medicinal product (CTIMP) and websites which listed complications and/or side effects for PILs associated with surgical trials. Supplementary file 1 contains a list of websites where this information was obtained.

### Data analysis

Descriptive statistics were presented using frequencies and percentages to report the usage of each principle in the PILs. Ratios indicate the number of PILs using the specific principle based on the total number of PILs. Percentages represent the proportion of PILs using each principle relative to the total number of PILs. Additionally, descriptive statistics are used to report the usage of the number of principles in each PIL. Percentages are rounded to the nearest 0.5%. A STROBE statement has been completed also.

## Results

Our sample included 214 PILs from 21 CTUs in England, Scotland, and Wales (of 34 registered in the UK and Northern Ireland) and all 5 of the CRFs operating at the time of data collection in the Republic of Ireland: 175 adult PILs (≥ 18 years) and 39 children PILs (< 18 years). Of the adult PILs, 134 were provided directly to adults regarding their own participation, 24 were provided to adults on behalf of a child, 8 were provided to obtain deferred consent, 5 were for legal representatives and 4 were provided to adults who had regained capacity. Of the children PILs, 17 were provided to teenagers, aged between 12 and 18 years, and various descriptions were used to describe the participants, e.g. older children, younger persons or the age was sometimes listed and ranged between 12 and 18 years. Twenty-two PILs were provided to children, < 12 years of age. The descriptions of the participants in this group included children, young children or school children. Sixty-five per cent of PILs (*n* = 140) were written between 2015 and 2021, 31% between 2010 and 2015 and the remainder (*n* = 8) prior to 2010. Ninety-two per cent of PILs were for academic trials. A full list of the characteristics of the PILs is shown in Table 1.

**Table 1:** Characteristics of Included PILs

	**Category**	**Sub-category**	***N* (214)**	**Per cent**
**Age**	**Adult (≥ 18 years)**	Standard adult PILs	134	63
		Adult representative on behalf of the child	24	11
		Deferred consent	8	4
		Legal representatives	5	2
		Regained capacity	4	2
		**Total adults**	**175**	**82**
	**Teenager (12–18 years)**	Teenager (ages 12–18)	17	8
	**Child (< 12 years)**	Child (< 12)	22	10
		**Total teenager/children**	**39**	**18**
**Year**		2003	1	0.5
		2004	1	0.5
		2007	2	1
		2008	1	0.5
		2009	3	1.5
		2010	3	1.5
		2011	3	1.5
		2012	11	5
		2013	8	4
		2014	23	11
		2015	18	8
		2016	32	15
		2017	34	16
		2018	40	19
		2019	12	5
		2020	15	7
		2021	7	3
**Clinical speciality**		Oncology	30	14
		Nephrology	23	11
		Neurology	19	9
		Obstetrics and gynaecology	18	8
		Dermatology	16	7
		Infectious diseases	15	7
		Cardiology	12	5
		Musculoskeletal	11	5
		Genetic condition	10	5
		Respiratory	8	3.5
		Otology	7	3
		Gastroenterology	6	3
		Neonatal care	6	3
		Vascular disease	6	3
		Immunology	5	2
		Chronic fatigue syndrome	5	2
		Public health	4	2
		Dental	4	2
		Intensive care	3	1
		Diabetes and endocrinology	2	1
		Haematology	2	1
		Ophthalmology	2	1
		Emergency care	1	0.5
		Mental health	1	0.5
		Sexual health	1	0.5
**Funding source**		Non-commercial	196	91.5
		Commercial	15	7
		Unclear (unable to determine from PIL alone)	3	1.5

Table 2 displays how the seven principles were used in the PILs. Principle 5 (all potential benefits and harms need to be compared with what would happen if the participant did not take part in the trial) and principle 7 (information regarding potential benefits and harms should not be presented apart by one or more pages) were used most frequently. Principles 2 (all potential harms of an intervention should be divided into serious and less serious) and 6 (suitable visual representations are recommended where appropriate to describe potential benefits and harms) were rarely used, and a small number (*n* = 13) used principle 3. Ninety-one per cent of PILs (*n* = 194) mentioned some harms associated with the intervention, of which 46% (*n* = 90) were drug trials and 35% (*n* = 67) were non-drug trials. Eighteen had no potential harms at all listed. In terms of principle 1 where all harms should be listed, only 27% did so. Forty-five per cent (*n* = 96) of PILs listed all potential benefits of the intervention (principle 4). Of the 54.5% (*n* = 117) that did not adhere to principle 4, 15% (*n* = 18)  did not list any benefits.

**Table 2:** Usage of the seven principles in the PILs principle

***N* = 214**		**Yes, *N* (%)**	**No, *N* (%)**	**N/A**^**a**^**, *N* (%)**	**Unclear**^**b**^**, *N* (%)**
Principle 1	All potential harms of an intervention should be listed	58 (27)	154 (72)	0 (0)	2 (1)
Principle 2	All potential harms should be divided into serious (life-threatening, causing permanent damage) and less serious (like a mild headache that goes away quickly)	3 (1)	185 (87)	26 (12)	0 (0)
Principle 3	It must be made explicit that not all potential harms are known	13 (6)	201 (94)	0 (0)	0 (0)
Principle 4	All potential benefits of the intervention should be listed	96 (45)	117 (54.5)	0 (0)	1 (0.5)
Principle 5	All potential benefits and harms need to be compared with what would happen if the participant did not take part in the trial	182 (85)	31 (14.5)	0 (0)	1 (0.5)
Principle 6	Suitable visual representations are recommended where appropriate to describe potential benefits and harms	1 (0.5)	0 (0)	0 (0)	213 (99.5)
Principle 7	Information regarding potential benefits and harms should not be presented apart by one or more pages	182 (85)	19 (9)	12 (5.5)	1 (0.5)

^a^Principle 2—N/A as no harms were listed; principle 7—N/A as there were either no harms or benefits listed

^b^Principle 1—unclear from the PIL what the study intervention was; principle 4—unclear because unclear what is intervention and what is standard of care; principle 5—unclear because no mention of standard care/usual care; principle 6—unclear as would need trial protocol to fully assess; principle 7—unclear as not clear what page benefits of intervention listed on

We calculated the proportion of PILs adhering to the range of principles from 0 to 7. Table 3 presents the results. The maximum number of principles adhered to was four. The most common number of principles adhered to was three (37.5%). Four per cent did not adhere to any principle. None of the PILs in our sample adhered to more than four principles.

**Table 3:** Number of principles adhered to

	*N* (214)	Per cent
Adhered to 0 principles	9	4
Adhered to 1 principle	22	10.5
Adhered to 2 principles	71	33
Adhered to 3 principles	80	37.5
Adhered to 4 principles	32	15
Adhered to 5 principles	0	0
Adhered to 6 principles	0	0
Adhered to 7 principles	0	0

## Discussion

### Summary of findings

We found that there is significant variation and inconsistency in the way potential trial benefits and harms are currently shared within PILs. This is true for both adult and children PILs and across disease areas.

### Comparison with other evidence

In a smaller sample, Kirby et al. found that 30% of PILs reported intervention benefits [10]. We found that when compared to potential risks, a minority of studies mentioned potential benefits (45% of PILs complied with principle 4 (all potential benefits should be listed)). Our results concurred with the Kirby et al. findings, which found that all PILs mentioned information about risks. We found that 90.5% (*n* = 194) of PILs in our much larger study mentioned potential harm(s) associated with the intervention. However, this figure is deceiving as only 27% (*n* = 58) of PILs presented information on *all* potential harms.

Our research also contributes to the large and growing body of evidence on the importance of placebo and nocebo effects (the effects of positive and negative expectations, respectively) [27]. The nocebo effect, which can be caused by over-emphasising harms and failing to mention potential benefits, has often been overlooked by clinicians and researchers [11, 13]. If future PILs adhere to the seven principles studied here, which insist that balanced information about potential benefits and harms be presented, it could result in lower rates of avoidable nocebo effects [28]. Also, harmonising the way PILs are designed promotes consistency. As a result, valuable time and effort could be saved in the creation and evaluation of PILs, ensuring a more efficient and standardised approach to conveying information about trial benefits and harms.

Our study identifies a potential contributor to drop-out rates and difficulties recruiting. By emphasising the information on harms (which we found was often presented before the information about potential benefits) [10], it could bias the participant to the potential for harm in the trial and result in low recruitment rates [29]. It could also cause participants to withdraw from trials early which can distort or complicate interpretation of results [30]. This withdrawal can introduce bias in meta-analyses, subsequently influencing decisions regarding the efficacy, efficiency and cost-effectiveness of treatments [13].

Our findings are relevant to ethical debates about informed consent. Participants have the right to be fully informed about the potential harms and benefits of their involvement in research and best practice is to do so: international standards demand it [7]. By failing to share information about potential benefits (where they exist), or exaggerating potential harms, the principle of non-maleficence could be violated [31].

## Strengths and limitations

A key strength of our study is the large number of PILs included which span a long period of time, and encompass a variety of trial designs, disease areas and funders. We requested any PILS that were available at the CTUs/CRFs; however, we are unsure how many were not provided. CTU/CRF directors could not share how many trials were open in their respective facilities. Additionally, when we requested the PILs, not all CTUs gave permission to share the PILs in an open-access paper and neither did they give permission to detail where the PILs originated. We were bound by the ethical approval for our study [26] to abide by these requests. Our study also had a number of other weaknesses. Determining whether some of the principles (especially principle 6) were adhered to depended on subjective judgement. For example, principle 6 discusses the appropriateness of visual representation. Appropriateness inevitably involves subjective judgement. Mitigating against this weakness, which the authors were aware of, judgements regarding appropriateness were made liberally, which resulted in the judgement about whether principle 6 was adhered to as usually ‘unclear’. This limitation mirrors a general problem in medical research regarding communicating risks to patients (either with words or visual representations) [32]. The process of contacting CRFs and CTUs to obtain PILs introduces the potential for selection bias. The willingness of these facilities to provide PILs may depend on their internal practices, resources or priorities. The method of gathering the PILs may also introduce availability bias and may only represent those PILs which were readily available and easily accessible. This can result in missing data or incomplete representation of PILs from certain trials or sponsors, leading to an incomplete picture of adherence to the principles. However, we believe our large sample size and representation from at least 26 different CTUs/CRFs mitigated this.

### Implications for practice

We recommend all CTUs/CRFs:

1. Rethink their PIL templates to include reference to the seven principles of good practice for describing potential benefits and harms in trials.
2. Standardise the presentation of trial benefits and harms in PILs.
3. Include patients and the public in the design of PILs to ensure they are relevant and acceptable. Though we did not assess the involvement of patients and the public in the design of the included PILs, we have made this recommendation previously in relation to disseminating trial results [33] and writing lay summaries [34]. Patients and the public should be involved in all stages of a trial from design to dissemination, including designing the PIL.

### Implications for future research

It is clear from multiple studies now that an agreed PIL template is needed for clinical trials. Funding to develop this and support international engagement will be necessary.

## Conclusion

The communication of trial harms and benefits in participant information leaflets (PILs) exhibits significant variability. Effectively communicating potential trial harms and benefits is crucial for informed consent and upholding the principles of autonomy. However, it is important to strike a balance by providing patients with appropriate information to prevent nocebo effects while respecting the ethical principles of respect for persons, beneficence and justice. Standardising the presentation of trial benefits and harms in PILs through the implementation of seven recently developed principles is an immediate first step in promoting transparency, consistency and reducing unwanted nocebo effects.

### Supplementary Information

**Additional file 1.** Websites and Information Referenced when assessing if PIL’s adhered to Principle 1 and Principle 4

**Additional file 2.**

## Data Availability

The datasets used and/or analysed during the current study are available from the corresponding author upon reasonable request.

## Abbreviations

CRF: Clinical Research Facility
CTIMP: Clinical trials of an investigational medicinal product
CTU: Clinical trial unit
GCP: Good Clinical Practice
ICF: Informed consent form
ICH GCP: International Conferences on Good Clinical Practice
MRC: Medical Research Council
OHRP: Office for Human Research Protections
PIL: Participant information leaflet
RCT: Randomised controlled trial
SpC: Summary of Product Characteristics
