# Supplementary material for: When describing harms and benefits to potential trial participants, participant information leaflets are inadequate
Source: Trials. 2024 May 1;25:292. doi: 10.1186/s13063-024-08087-9 (PMC11061982; doi:10.1186/s13063-024-08087-9)
Supplement: Supplementary file 1 — Additional file 1. Websites and Information Referenced when assessing if PIL’s adhered to Principle 1 and Principle 4 [file 13063_2024_8087_MOESM1_ESM.docx]

# **Supplementary File 1**

*Websites and Information Referenced when assessing if PIL’s adhered to Principle 1 and Principle 4*

**Adult PIL’s**

PIL 5 MifeMiso Trial- Reviewed SPMC for misoprostol [Angusta 25 microgram tablets - Summary of Product Characteristics (SmPC) - (emc) (medicines.org.uk)](https://www.medicines.org.uk/emc/product/12147/smpc) and

PIL 5 Mifemiso Trial- When assessing benefits reviewed study protocol [MifeMiso trial - University of Birmingham](https://www.birmingham.ac.uk/research/bctu/completed-trial-research/womens/mifemiso/index.aspx)

Pil 13, SpC of Gabapentin reviewed when decided re: harms and benefits.
[Licence_PA2315-164-005_18072022124414.pdf (hpra.ie)](https://www.hpra.ie/img/uploaded/swedocuments/Licence_PA2315-164-005_18072022124414.pdf)

PIL 19 deciding if all risks outlined foy myomectomy Mayo Clinic accessed at <https://www.mayoclinic.org/tests-procedures/myomectomy/about/pac-20384710> and Cleveland Clinic accessed at <https://my.clevelandclinic.org/health/treatments/15448-myomectomy>

PIL 19 deciding if all risks outlined for UAE accessed at <https://my.clevelandclinic.org/health/treatments/17954-uterine-artery-embolization>

PIL 23 SpC for progesterone accessed at [Utrogestan 100mg Capsules - Summary of Product Characteristics (SmPC) - (emc) (medicines.org.uk)](https://www.medicines.org.uk/emc/product/352/smpc)

PIL 24 SpC for digoxin and bisoprolol accessed when deciding re: potential harms

PIL 29 SpC for levothyroxine accessed when deciding re principle 1 at [Levothyroxine 100mcg tablets - Summary of Product Characteristics (SmPC) - (emc) (medicines.org.uk)](https://www.medicines.org.uk/emc/product/5682/smpc)

Pil 35 SpC for Mylotarg accessed when deciding re principle 1 accessed at [MYLOTARG 5mg powder for concentrate for solution for infusion | SPC | Medicines.ie](https://www.medicines.ie/medicines/mylotarg-5mg-powder-for-concentrate-for-solution-for-infusion-34452/spc#tabs)

Pil 36 SpC for Mylotarg accessed when deciding re principle 1 accessed at [MYLOTARG 5mg powder for concentrate for solution for infusion | SPC | Medicines.ie](https://www.medicines.ie/medicines/mylotarg-5mg-powder-for-concentrate-for-solution-for-infusion-34452/spc#tabs)

Pil 40 SpC for Mycophenolate accessed when deciding re principle 1 accessed at <https://www.medicines.org.uk/emc/product/1103/smpc#gref>

PIL 41 SpC for Fragmin accessed when decided on Principle 1, accessed at [Fragmin 5000 IU solution for injection - Summary of Product Characteristics (SmPC) - (emc) (medicines.org.uk)](https://www.medicines.org.uk/emc/product/4247/smpc)

Pil 47 SpC for local anaesthetic when deciding on Principle 1 accessed at <https://www.hpra.ie/img/uploaded/swedocuments/LicenseSPC_PA0196-013-002_21062017130043.pdf>

PiL 49 When deciding on Principle 1 regarding self management of Pessary insertion information [PIL Self Managament FAQ.pdf (ukcs.uk.net)](https://www.ukcs.uk.net/resources/Documents/Pessary%202021/PIL%20Self%20Managament%20FAQ.pdf)

PIL 58 When deciding on Principle 1 regarding risks associated with drug, SpC references accessed [Hexvix powder and solvent for solution for intravesical use ENG SmPC_09001bee807a34b4.pdf (mpa.se)](https://docetp.mpa.se/LMF/Hexvix%20powder%20and%20solvent%20for%20solution%20for%20intravesical%20use%20ENG%20SmPC_09001bee807a34b4.pdf)

PIL 60 Cleveland Clinic used to determine if all risks listed for both procedures
[Ureteroscopy + kidney stones (clevelandclinic.org)](https://my.clevelandclinic.org/health/treatments/16213-ureteroscopy#:~:text=Other%20side%20effects%20include%20cramps%20in%20the%20kidney,symptoms%20may%20last%20until%20the%20stent%20is%20removed.)
[Shock Wave Lithotripsy (Kidney Stone Treatment): Procedure Details & Recovery (clevelandclinic.org)](https://my.clevelandclinic.org/health/treatments/16582-shockwave-lithotripsy#risks--benefits)

PIL 65 trabeculectomy risks [Trabeculectomy: Complications, Success Rate, and More (healthline.com)](https://www.healthline.com/health/trabeculectomy#risk)

PIL 70 Cancer research UK information regarding gemcitabine [Gemcitabine (Gemzar) | Cancer information | Cancer Research UK](https://www.cancerresearchuk.org/about-cancer/treatment/drugs/gemcitabine)

PIL 71 Spc of HCQ to determine response for Principle 1 [Plaquenil Tablets | SPC | Medicines.ie](https://www.medicines.ie/medicines/plaquenil-tablets-33380/spc)

PIL 72 Spc of Rituximab to determine response for Principle 1 [MabThera 100 mg and 500 mg concentrate for solution for infusion | SPC | Medicines.ie](https://www.medicines.ie/medicines/mabthera-100-mg-and-500-mg-concentrate-for-solution-for-infusion-32771/spc)

PIL 77 NHS Website on BCG vaccine side effects [BCG (TB) vaccine side effects - NHS (www.nhs.uk)](https://www.nhs.uk/conditions/vaccinations/bcg-tb-vaccine-side-effects/)

PIL 78 Dexemethasone side effects [Dexamethasone 2mg Tablets | SPC | Medicines.ie](https://www.medicines.ie/medicines/dexamethasone-2mg-tablets-31841/spc#!)

Pil 80 Telmisartan Spc [Telmisartan Mylan 20mg 40mg 80mg Tablets | SPC | Medicines.ie](https://www.medicines.ie/medicines/telmisartan-mylan-20mg-40mg-80mg-tablets-33960/spc)

Pil 85 Lamotrigine Spc [Lamictal 25mg tablets | SPC | Medicines.ie](https://www.medicines.ie/medicines/lamictal-25mg-tablets-32631/spc#tabs)

Pil 86 Iron infusion Ferinject Spc [Ferinject (ferric carboxymaltose) | SPC | Medicines.ie](https://www.medicines.ie/medicines/ferinject-ferric-carboxymaltose--32142/spc)

Pil 92 Co-Amoxiclav SpC [PACKAGE LEAFLET (hpra.ie)](https://www.hpra.ie/img/uploaded/swedocuments/bcf9a86f-a9ea-4205-a353-07d16d791d45.pdf)

Pil 95 Ursofalk SpC [Ursofalk 250mg Hard Capsules | SPC | Medicines.ie](https://www.medicines.ie/medicines/ursofalk-250mg-hard-capsules-34113/spc)

PIL 96 laropiprant and nicotinic acid combined in Trevaclyn [Trevaclyn (clone Tredaptive), INN-nicotinic acid/laropiprant (europa.eu)](https://www.ema.europa.eu/en/documents/product-information/trevaclyn-epar-product-information_en.pdf)

PIL 98 Fenofibrate SpC [Lipantil Micro 200mg capsules, hard | SPC | Medicines.ie](https://www.medicines.ie/medicines/lipantil-micro-200mg-capsules-hard-32699/spc)

Pil 100 Azithromyic SpC [Licence_PA0822-191-001_12012021150540.pdf (hpra.ie)](https://www.hpra.ie/img/uploaded/swedocuments/Licence_PA0822-191-001_12012021150540.pdf)

PIL 101 Humira SpC [Humira 40mg solution for injection in pre-filled pen | SPC | Medicines.ie](https://www.medicines.ie/medicines/humira-40mg-solution-for-injection-in-pre-filled-pen-32384/spc)

PIL 107 Clarithromycin Spc [Klacid 250mg Film-coated Tablets | SPC | Medicines.ie](https://www.medicines.ie/medicines/klacid-250mg-film-coated-tablets-32603/spc)

Pil 112 Exparel local anaesthetic [Exparel, INN- bupivacaine (europa.eu)](https://www.ema.europa.eu/en/documents/product-information/exparel-liposomal-epar-product-information_en.pdf)

PIL 113 Methotrexate SpC [Methotrexate 2.5mg Tablets | SPC | Medicines.ie](https://www.medicines.ie/medicines/methotrexate-2-5mg-tablets-32839/spc)

PIL 114 Dosycycline SpC [Vibramycin Capsules 100mg | SPC | Medicines.ie](https://www.medicines.ie/medicines/vibramycin-capsules-100mg-34182/spc)

PIL 115 Bexsero Meningococcoal vaccine [Bexsero, common name - meningococcal group B Vaccine (rDNA, component, adsorbed) (europa.eu)](https://www.ema.europa.eu/en/documents/product-information/bexsero-epar-product-information_en.pdf)

PIL 120 BCG Medac SpC [8f5d879a-0040-4b13-a7f3-43f5a3f49a4b.pdf (hpra.ie)](https://www.hpra.ie/img/uploaded/swedocuments/8f5d879a-0040-4b13-a7f3-43f5a3f49a4b.pdf)

PIL 122 Tamoxifen SpC [ie-spc-pr2671394rtq-clean.pdf](about:blank)

PIL 127 Mesothelioma Center Page re: side effects of radiation [Radiation Therapy for Mesothelioma: Is It Right for You? (asbestos.com)](https://www.asbestos.com/treatment/radiation/#:~:text=Side%20Effects%20of%20Radiation%20Therapy%201%20Skin%20problems,pneumonitis%29%207%20Scarring%20of%20the%20lungs%20%28radiation%20fibrosis%29)

PIL 130 Lyrica SPc [LYRICA Capsules | SPC | Medicines.ie](https://www.medicines.ie/medicines/lyrica-capsules-32756/spc)

PIL 134 Complications of hip arthroscopy procedure [Complications in Hip Arthroscopy - PMC (nih.gov)](https://www.ncbi.nlm.nih.gov/pmc/articles/PMC5193532/)

**All Other PILs**

Pil 1- Eculizumab SpC <https://www.medicines.ie/medicines/soliris-33776/spc>

Pil 2- Eculizumab SpC https://www.medicines.ie/medicines/soliris-33776/spc

Pil 3- Eculizumab SpC https://www.medicines.ie/medicines/soliris-33776/spc

Pil 4- Eculizumab SpC <https://www.medicines.ie/medicines/soliris-33776/spc>

PIL 8- Prednisolone SpC <https://www.medicines.ie/medicines/prednisolone-5mg-soluble-tablets-34744/spc>

PIL 15 Azithromycin SPC [Azithromycin: MedlinePlus Drug Information](https://medlineplus.gov/druginfo/meds/a697037.html)

PIL 40 Fibrinogen SpC [pil.10315.pdf (medicines.org.uk)](https://www.medicines.org.uk/emc/files/pil.10315.pdf)

PIL 41 Dexamethasone SpC [Dexa_Tab_IE_S_2mg_v8 (1).pdf](about:blank)

PIL 42 Rosuvastatin [Rosuvastatin Mylan 5mg 10mg 20mg & 40mg Film-coated Tablets | SPC | Medicines.ie](https://www.medicines.ie/medicines/rosuvastatin-mylan-5mg-10mg-20mg-40mg-film-coated-tablets-33654/spc)

PIL 43 Rosuvastatin [Rosuvastatin Mylan 5mg 10mg 20mg & 40mg Film-coated Tablets | SPC | Medicines.ie](https://www.medicines.ie/medicines/rosuvastatin-mylan-5mg-10mg-20mg-40mg-film-coated-tablets-33654/spc)

PIL 44 Rosuvastatin [Rosuvastatin Mylan 5mg 10mg 20mg & 40mg Film-coated Tablets | SPC | Medicines.ie](https://www.medicines.ie/medicines/rosuvastatin-mylan-5mg-10mg-20mg-40mg-film-coated-tablets-33654/spc)

PIL 45 Rosuvastatin [Rosuvastatin Mylan 5mg 10mg 20mg & 40mg Film-coated Tablets | SPC | Medicines.ie](https://www.medicines.ie/medicines/rosuvastatin-mylan-5mg-10mg-20mg-40mg-film-coated-tablets-33654/spc)

PIL 46 Rosuvastatin [Rosuvastatin Mylan 5mg 10mg 20mg & 40mg Film-coated Tablets | SPC | Medicines.ie](https://www.medicines.ie/medicines/rosuvastatin-mylan-5mg-10mg-20mg-40mg-film-coated-tablets-33654/spc)

PIL 47 Lamotrigine [Lamictal 25mg tablets | SPC | Medicines.ie](https://www.medicines.ie/medicines/lamictal-25mg-tablets-32631/spc)

PIL 48 Lamotrigine [Lamictal 25mg tablets | SPC | Medicines.ie](https://www.medicines.ie/medicines/lamictal-25mg-tablets-32631/spc)

PIL 49 Lamotrigine [Lamictal 25mg tablets | SPC | Medicines.ie](https://www.medicines.ie/medicines/lamictal-25mg-tablets-32631/spc)

PIL 50 Lamotrigine [Lamictal 25mg tablets | SPC | Medicines.ie](https://www.medicines.ie/medicines/lamictal-25mg-tablets-32631/spc)

PIL 51 Lamotrigine [Lamictal 25mg tablets | SPC | Medicines.ie](https://www.medicines.ie/medicines/lamictal-25mg-tablets-32631/spc)

PIL 55 Tobramycin [Tobramycin 40 mg/ml Solution for Injection | SPC | Medicines.ie](https://www.medicines.ie/medicines/tobramycin-40-mg-ml-solution-for-injection-33996/spc#!)

PIL 56 Ciclosporin Spc [Licence_PA0896-024-001_09012023152416.pdf (hpra.ie)](https://www.hpra.ie/img/uploaded/swedocuments/Licence_PA0896-024-001_09012023152416.pdf)

PIL 57 Ciclosporin SpC [Licence_PA0896-024-001_09012023152416.pdf (hpra.ie)](https://www.hpra.ie/img/uploaded/swedocuments/Licence_PA0896-024-001_09012023152416.pdf)

PIL 58 Ciclosporin SpC [Licence_PA0896-024-001_09012023152416.pdf (hpra.ie)](https://www.hpra.ie/img/uploaded/swedocuments/Licence_PA0896-024-001_09012023152416.pdf)

PIL 59 Ciclosporin SpC [Licence_PA0896-024-001_09012023152416.pdf (hpra.ie)](https://www.hpra.ie/img/uploaded/swedocuments/Licence_PA0896-024-001_09012023152416.pdf)

PIL 61 Tranxemic Acid SpC [Licence_PA2010-055-001_30112020112620.pdf (hpra.ie)](https://www.hpra.ie/img/uploaded/swedocuments/Licence_PA2010-055-001_30112020112620.pdf)

PIL 67 Ibruofen SpC [Ibuprofen 200mg Soft Capsules | SPC | Medicines.ie](https://www.medicines.ie/medicines/ibuprofen-200mg-soft-capsules-31215/spc)

PI; 68 Lectoferrin SpC [License (hpra.ie)](https://www.hpra.ie/img/uploaded/swedocuments/LicenseSPC_PA0949-003-002_09062016144043.pdf)

PIL 70 Spc Morphine [Licence_PA0073-020-001_22032021185627.pdf (hpra.ie)](https://www.hpra.ie/img/uploaded/swedocuments/Licence_PA0073-020-001_22032021185627.pdf)

PIL 74 Co-Amoxiclav SpC [Germentin 250mg/125mg Film-coated Tablets | SPC | Medicines.ie](https://www.medicines.ie/medicines/germentin-250mg-125mg-film-coated-tablets-32282/spc)
